# Preimplantation genetic testing and prenatal diagnosis in a family with pseudovaginal perineoscrotal hypospadias: A case report

**DOI:** 10.1097/MD.0000000000036171

**Published:** 2023-11-17

**Authors:** Jiayao Chen, Zhiping Zhang, Wenjing Shi, Qin Yan, Xingyu Bi, Pengfei Zhu, Dongdong Zhang, Xueqing Wu

**Affiliations:** a Shanxi Medicine University, Taiyuan, Shanxi, China; b Children’s Hospital of Shanxi Medicine University, Taiyuan, Shanxi, China; c Center of Reproductive Medicine, Children’s Hospital of Shanxi and Women Health Center of Shanxi, Taiyuan, Shanxi, China.

**Keywords:** preimplantation genetic testing, prenatal diagnosis, pseudovaginal perineoscrotal hypospadias, *SRD5A2* gene

## Abstract

**Rationale:**

Pseudovaginal perineoscrotal hypospadias (PPSH) is a rare autosomal recessive disorder of sex development caused by biallelic mutations in *SRD5A2*. PPSH is characterized by a vaginal-like blind ending perineal opening, penoscrotal hypospadias, and impaired masculinization.

**Patient concerns:**

We reported preimplantation genetic testing and prenatal diagnosis in a family with PPSH.

**Diagnosis:**

Whole-exome sequencing of the family identified 2 *SRD5A2* pathogenic variants (c.578A>G and c.607G>A). Haplotype analysis showed that the variants were inherited from the previous generation of this family.

**Interventions:**

During subsequent in vitro fertilization, preimplantation genetic testing was performed on 9 embryos. One unaffected embryo was transferred, resulting in a singleton pregnancy.

**Outcomes:**

The prenatal diagnosis at 20 weeks’ gestation confirmed the fetus was unaffected. A healthy female infant weighing 3100 g and measuring 50 cm was delivered vaginally at 39^+5^ weeks of gestation.

**Lessons subsections:**

This case highlights the use of preimplantation genetic testing and prenatal diagnosis to prevent the transmission of PPSH in families at risk. Our approach provides an effective strategy for identification and management of families with autosomal recessive disorders like PPSH.

## 1. Introduction

Pseudovaginal perineoscrotal hypospadias (PPSH) is an autosomal recessive disorder of sex development caused by mutations in *SRD5A2*. The mutations in the *SRD5A2* gene results in a failure to convert testosterone to dihydrotestosterone, resulting in normal or high serum concentrations of testosterone for males, but a low level of dihydrotestosterone (DHT) and an increased T/DHT ratio at baseline or after human chorionic gonadotropin (HCG) stimulation,^[1]^ thus producing a wide range of genital ambiguity at birth.^[2]^ Patients with PPSH exhibit impaired masculinization and a spectrum of genital anomalies ranging from predominantly female to ambiguous.^[3]^ Preimplantation genetic testing for monogenic disorders (PGT-M) allows for identification of pathogenic variants in embryos so that unaffected embryos can be selected for transfer, preventing transmission of genetic diseases to offspring.^[4,5]^ While surgical treatment of genital anomalies associated with *SRD5A2* variants is well studied, less is known about the use of PGT-M to prevent *SRD5A2*-related disorders. We identified a family carrying *SRD5A2* variants. With informed consent, we performed PGT-M in this family and successfully prevented transmission of PPSH to the next generation.

## 2. Materials and methods

### 2.1. Clinical data

A married couple with a history of adverse pregnancy outcomes presented to our reproductive medicine center in October 2021 requesting preimplantation genetic testing (PGT) to prevent recurrence in future pregnancies. They had 2 sons with disorders of sex development, hypospadias, and neonatal death in 2017 and 2019, respectively. Comprehensive medical evaluations did not reveal the etiology. With informed consent from the couple and their families, we performed whole-exome sequencing (WES) (without proband), PGT-M and prenatal diagnosis.

### 2.2. Research methods

#### 2.2.1. Pathogenic variant detection.

Genomic DNA was extracted from 5 mL peripheral blood samples (Blood Genome Column Medium Extraction Kit (Kangweishiji, China)) from the couple and their respective parents. Whole-exome sequencing was performed to detect pathogenic variants, followed by Sanger sequencing validation of detected variants in all samples.

#### 2.2.2. Family haplotyping.

To determine the parental origin of the pathogenic variants, single nucleotide polymorphism (SNP) genotyping was performed using the Illumina Infinium Asian Screening Array-24 v1.0 Kit. Genomic DNA samples from the couple and their parents were analyzed to establish SNP-linked haplotypes. The haplotype co-segregating with the pathogenic variants was identified for subsequent embryo haplotyping during PGT.

#### 2.2.3. Preimplantation genetic testing and IVF.

Following haplotyping, PGT-M and IVF were performed. A long gonadotropin-releasing hormone agonist protocol was used for controlled ovarian stimulation. 28 oocytes were retrieved, of which 18 metaphase II (MII) oocytes underwent intracytoplasmic sperm injection. Embryos were cultured to the blastocyst stage on day 5. Trophectoderm biopsies were performed on 9 morphologically suitable blastocysts for genetic testing. Biopsied blastocysts were vitrified after biopsy.

#### 2.2.4. Single-cell whole genome amplification and sequencing.

Trophectoderm biopsy samples were subjected to whole genome amplification (WGA) using the ChromSwiftTM kit (Yikon Genomics, China) according to the manufacturer’s instructions. WGA products were fragmented and libraries were constructed for next-generation sequencing on the Illumina Nextseq 550 platform to analyze embryonic ploidy. The WGA products also underwent SNP haplotyping and Sanger sequencing of the pathogenic variants to determine the embryo genotype. Embryos without the familial pathogenic variants and normal chromosomal ploidy were selected for transfer based on the comprehensive analysis.

#### 2.2.5. Embryo transfer and follow-up.

Twelve days after embryo transfer, the female’s serum HCG level was 455.96 mIU/mL. Four weeks after transfer, a transvaginal ultrasound showed an intrauterine singleton pregnancy, confirming a clinical pregnancy. At 20 weeks of gestation, amniocentesis and fetal ultrasound were performed to determine fetal ploidy and genotype.

## 3. Results

### 3.1. The couple carried pathogenic variants in *SRD5A2*

**Figure 1.**
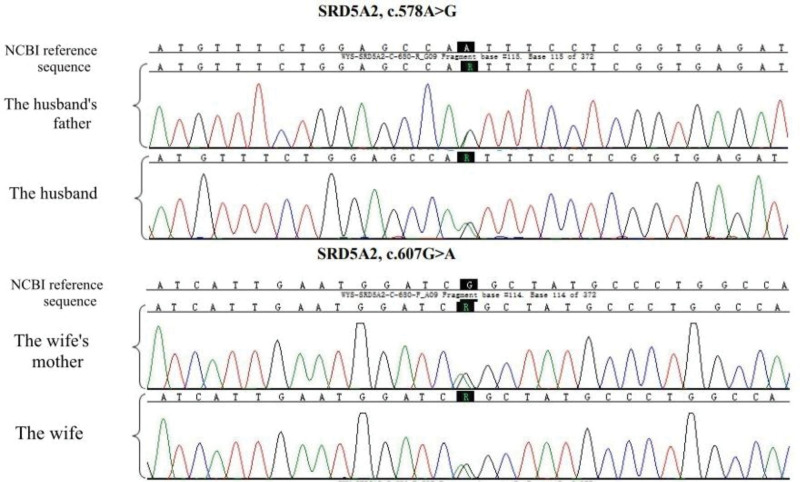
The couple and their families carried pathogenic variants in *SRD5A2.*

**Figure 2.**
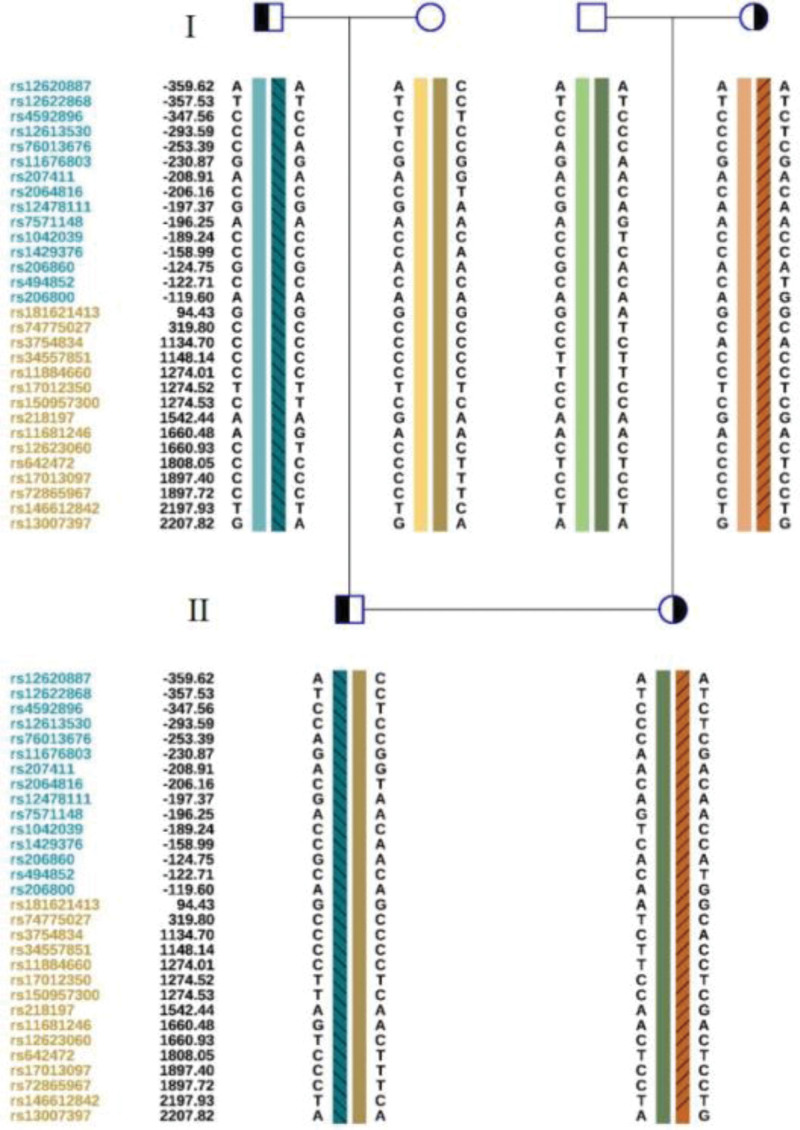
Family lineage *SRD5A2* gene family map and results of SNP typing linkage analysis in family pre-experiment. SNP = single nucleotide polymorphism.

The couple underwent WES, followed by Sanger sequencing validation of detected variants in their respective parents. The husband and his father harbored the *SRD5A2* c.578A>G pathogenic variant. The wife and her mother harbored the *SRD5A2* c.607G>A pathogenic variant (Fig. 1). Thus, the couple both carried heterozygous pathogenic variants in *SRD5A2*, the gene associated with PPSH. This was consistent with the phenotypic manifestations observed in their deceased sons. The husband’s mother and wife’s father did not carry pathogenic/likely pathogenic variants in this gene (Fig. 2).

### 3.2. PGT-M and pregnancy outcome

The couple was informed of the risk of re-occurrence in the offspring after genetic counseling. They requested PGT-M to assist in pregnancy. In the PGT-M family pre-experiment, SNP haplotyping was performed, and consistent with the gene testing results, the pathogenic haplotype was derived from both sexes (Fig. 2).

**Figure 3.**
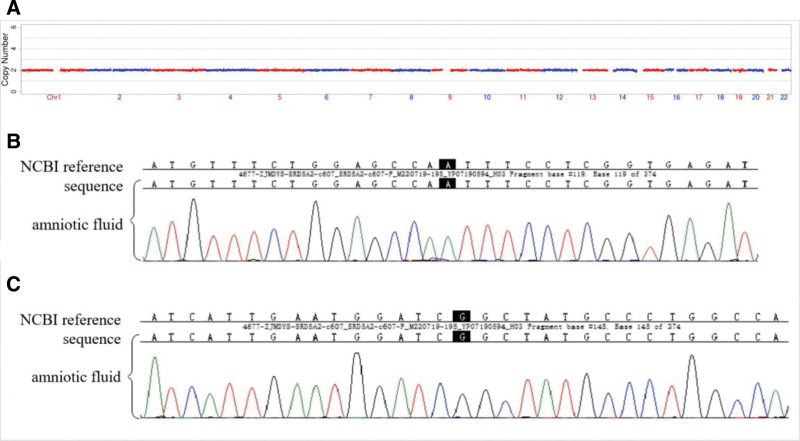
Amniocentesis results at 20 weeks of gestation: (A) fetal amniotic fluid exfoliation cell CNV results; ultrasound-guided amniocentesis at 20 weeks of gestation suggest: (B) no c.578A>G mutations; (C) no c.607G>A mutations. CNV = copy number variation.

Haplotyping confirmed that the pathogenic variants originated from both the husband and wife (Fig. 2). Trophectoderm biopsies from 9 morphologically suitable blastocysts underwent successful WGA. Analysis of the 9 embryos revealed that 5 were euploid without the paternal or maternal pathogenic *SRD5A2* variants (Table 1). Following genetic counseling, a frozen-thawed transfer of embryo 31636_2 was performed in February 2022.Twelve days after transfer, the serum HCG was 455.96 mIU/mL. Ultrasound 4 weeks after transfer showed an ongoing singleton intrauterine pregnancy. Amniocentesis at 20 weeks of gestation revealed a normal karyotype and negative results for copy number variations (Fig. 3A). No pathogenic *SRD5A2* variants were detected (Fig. 3B and C). The remaining prenatal evaluations were normal. A healthy female infant weighing 3100 g and measuring 50 cm was delivered vaginally at 39^+5^ weeks of gestation. After delivering a healthy fetus, patients and their families are very happy and approval our treatment options.

**Table 1:** Results of 9 blastocyst biopsy samples.

Sample name	Morphology score	CNV test results	SNP chain analysis Judgment results	Mutation site detection results	
				*SRD5A2*, c.578A>G	*SRD5A2*, c.607G>A
31636_1	5BB	46, XN	Carrying *SRD5A2* gene c.607G>A heterozygous Mutation (maternal origin)	No mutation	Heterozygous mutation
31636_2	5AB	46, XN	Not carrying the *SRD5A2* gene c.578A>G and c.607G>A mutations	No mutation	No mutation
31636_3	5BB	46, XN	Not carrying the *SRD5A2* gene c.578A>G and c.607G>A mutations	No mutation	No mutation
31636_4	5AB	46, XN	Carrying *SRD5A2* gene c.607G>A heterozygous Mutation (maternal origin)	No mutation	Heterozygous mutation
31636_5	5AB	46, XN	Not carrying the *SRD5A2* gene c.578A>G and c.607G>A mutations	No mutation	No mutation
31636_6	5BB	46, XN	Carrying the *SRD5A2* gene c.578A>G heterozygous mutation (paternal origin)	Heterozygous mutation	No mutation
31636_7	5AB	46, XN	Not carrying the *SRD5A2* gene c.578A>G and c.607G>A mutations	No mutation	No mutation
31636_8	5AB	46, XN	Not carrying the *SRD5A2* gene c.578A>G and c.607G>A mutations	No mutation	No mutation
31636_9	5BC	46, XN	Carrying the *SRD5A2* gene c.578A>G heterozygous mutation (paternal origin)	Heterozygous mutation	No mutation

SNP = single nucleotide polymorphism.

## 4. Discussion

Pseudovaginal perineoscrotal hypospadias (PPSH) results from deficiency of steroid 5-α reductase type 2, an enzyme that catalyzes the conversion of T to DHT using NADPH as a cofactor.^[6]^ Variants in *SRD5A2*, the gene encoding this enzyme, lead to impaired conversion of T to DHT, resulting in normal or high T levels but low DHT levels and an increased T/DHT ratio at baseline or after HCG stimulation.^[1]^ This produces a spectrum of genital ambiguity at birth, though testosterone and dihydrotestosterone levels do not strongly correlate with phenotypic severity.^[7]^

The *SRD5A2* gene on chromosome 2p23.1 contains 5 exons and 4 introns, encoding a 254-amino acid protein with an androgen-binding domain (N-terminal)) and NADPH cofactor -binding domain (C-terminal).^[1]^ It is abundant in embryonic testis and prostate tissue. Most variants occur in exon 1 and exon 4, considered hotspots.^[1]^ Reported variant types include missense, nonsense, splice site, frameshift, and large deletions, with over 200 variants in ClinVar and 186 in HGMD.^[8,9]^ Here, the couple had the common p.G203S variant, causing a glycine to serine change at residue 203 in the transmembrane domain, and the rare p.D193S variant, changing aspartic acid to serine at residue 193. The p.G203S variant reduces enzyme activity by ~60% and correlates with moderate to severe phenotypes,^[7,8]^ while the p.D193S variant has not been functionally characterized. In general, loss-of-function variants correlate with severe phenotypes, while some residual activity correlates with variable, milder phenotypes, but other factors also contribute.^[8]^

There are about 7000 monogenic disorders, most without effective treatment, creating substantial burdens.^[10]^ Preimplantation genetic testing (PGT) screens embryos before transfer, allowing selection of unaffected embryos.^[4,5]^ PGT-M prevents pregnancy termination and associated trauma by identifying affected embryos preimplantation.^[5,11]^ With improving technology, PGT-M prevents genetic disease transmission and birth of affected children.^[6]^

PGT-M requires genetic testing, often using whole genome amplification, SNP analysis, and next-generation sequencing to identify variants.^[6,12,13]^ Before PGT-M, genetic counseling educates couples about the condition and options, allowing informed decisions.^[14]^ Single embryo transfer is recommended after PGT-M.^[14]^

Though technology has improved, with <1% misdiagnosis risk, PGT-M remains a screening method.^[15,16]^ Errors can occur due to technical, biological and human factors. Chinese guidelines recommend prenatal diagnosis after PGT-M to confirm results and avoid adverse outcomes or transmission of pathogenic variants missed by PGT-M.^[13]^ Noninvasive prenatal testing is not currently recommended.^[17]^ While prenatal diagnosis also has limitations, informing families allows maximum prevention of transmission and achievement of eugenic goals.^[17]^

Here, we used WES to identify SRD5A2 variants in a family with PPSH, enriching the variant spectrum and providing a precise tool for carriers seeking pregnancy, and achieved a high level of patient satisfaction. We recommend PGT-M and prenatal diagnosis for couples with adverse pregnancy history to prevent familial transmission of disease-causing variants.

## Acknowledgments

We would like to thank the patients for participating in this study.

## Author contributions

**Investigation:** Wenjing Shi.

**Methodology:** Qin Yan.

**Project administration:** Xingyu Bi.

**Resources:** Pengfei Zhu.

**Supervision:** Xueqing Wu.

**Validation:** Dongdong Zhang.

**Writing – original draft:** Jiayao Chen.

**Writing – review & editing:** Jiayao Chen, Zhiping Zhang.
